# Long-term patterns of adherence to medication therapy among patients with type 2 diabetes mellitus in Denmark: The importance of initiation

**DOI:** 10.1371/journal.pone.0179546

**Published:** 2017-06-30

**Authors:** Majken Linnemann Jensen, Marit Eika Jørgensen, Ebba Holme Hansen, Lise Aagaard, Bendix Carstensen

**Affiliations:** 1 Steno Diabetes Center Copenhagen, Gentofte, Denmark; 2 Faculty of Health and Medical Sciences, Department of Pharmacy, Section for Social and Clinical Pharmacy, Universitetsparken 2, University of Copenhagen, Denmark; 3 Faculty of Health, University of Southern Denmark, Odense, Denmark; Kaohsiung Medical University Hospital, TAIWAN

## Abstract

**Aims:**

Poor adherence to medication therapy among type 2 diabetes patients is a clinical challenge. We aimed to determine which factors are associated with the three phases of long-term adherence to medication: initiation, implementation and discontinuation in a register-based study.

**Methods:**

Adherence to six medicine groups (metformin, sulfonylureas, acetylsalicylic acid, thiazide diuretics, renin angiotensin system inhibitors, and statins) were analysed among 5,232 patients with type 2 diabetes at a tertiary referral hospital during 1998–2009. Rate-ratios of initiation of treatment, recurrent gaps in supply of medication, and discontinuation of treatment were analysed using Poisson regression.

**Results:**

Poor initiation rather than poor implementation or discontinuation was the main contributor to medication nonadherence. Polypharmacy was a risk factor for slower initiation of treatment for all six medicine groups (rate ratio ranging 0.79 95%CI [0.72–0.87] to 0.89 95%CI [0.82–0.96] per already prescribed medicine), but once patients were in treatment, polypharmacy was not associated with recurrence of gaps in supply of medication, and polypharmacy was associated with lower risk of discontinuation (rate ratio ranging 0.93 95%CI [0.86–1.00] to 0.96 95%CI [0.93–0.99] per prescribed medicine). Other identified risk factors for slow initiation, poor implementation, and discontinuation were diabetes duration, younger age, and Turkish/Pakistani origin.

**Discussion:**

This study showed that a risk factor does not necessarily have the same association with all three elements of adherence (initiation, implementation and discontinuation), and that efforts supporting patients introduced to more complex drug combinations should be prioritized.

## Introduction

Poor adherence to medication therapy in chronic disease is associated with a worsening of clinical status/health[1, 2], higher risk of hospitalisation[3], risk of medicine-related hospital admissions[4] and higher mortality risk[5]. Losing adherence has been shown to be associated with more hospitalizations and emergency department visits[6]. Improving adherence to medicine could be a more efficient strategy with higher impact than any treatment itself[7].

Patients with type 2 diabetes mellitus (T2DM) are often challenged by a complex medicine regimen targeting multiple cardiovascular disease risk factors[8, 9]. Many efforts have been made to describe the potential predictors of adherence[10, 11][12,13]. Recent research has focused on three different phases of adherence to medication: initiation, implementation, and discontinuation of medication therapy[12–17], but it has primarily focused on short-term studies with follow-up of less than three years. If and how soon patients initiate treatment after being prescribed with medication is of interest, as well as how well patients implement the dosing regimen afterwards. In chronic care, there is risk of occasionally running out of supplies of medicine, both intentional and unintentional. Tracking events of running out of supply of medicine, here referred to as gaps in supply of medication, and tracking the length of these gaps provide information on implementation of the prescribed medication therapy. Finally, tracking if patients discontinue treatment early is also of importance. Examining the associations between predictors of adherence and the three different components of adherence: initiation, implementation, and discontinuation of medication therapy, may be beneficial for patient-specific initiatives to improve adherence to medicine in diabetes.

Our aim was to estimate rates of initiation of medication therapy, the pattern of recurrent gaps in supply of medication as a measure of implementation, and early discontinuation of therapy for frequently used medicines among Danish patients with T2DM using a new method and algorithm[18] for calculation of adherence to medication. Furthermore, we aimed to investigate how condition-, therapy-, and patient-related factors are associated with the three different phases of adherence to medication: initiation, implementation and discontinuation among this group of T2DM patients.

## Methods

### Setting

The study was conducted among T2DM patients referred to the outpatient clinic at Steno Diabetes Center (Steno) during 1998–2009 and prescribed at least once with glucose lowering, antihypertensive, lipid lowering or anti-coagulation therapy. Steno is a specialised diabetes tertiary referral hospital for both type 1 and T2DM patients. Typically, T2DM patients with poor glycaemic control are referred to Steno from general practice for a shorter time period in order to improve glucose control or cardiovascular disease risk factors before they are discharged back to general practice. In addition, T2DM patients with complications are referred to Steno for permanent diabetes treatment and control. Patients were followed from 1 January 1998 or date of first entry at Steno after 1 January 1998 to the earliest of a) 31 December 2009, b) the latest date of referral of the patient from Steno to general practice or another specialist unit, or c) date of death. Time spent enrolled in clinical trials at Steno, which might have included interventions with medicine/placebo, or temporarily referred away from Steno during follow-up was censored. Follow-up time was split in intervals of 3 months allowing the time-varying covariates to alter with the same frequency using the value of the left endpoint of the 3 months intervals.

### Outcome

We studied four different adherence outcomes of clinical interest: (**A**) **initiation of medication** therapy, where the patient is in a state of waiting from the time the medicine is prescribed until it is redeemed for the first time. After initiation the patient enters a state of being in treatment (persistence), and the patient can only adhere to the prescribed medication if he or she fill the prescription frequently enough to allow for a sufficient supply of the drug. If the patient runs out of supply before refilling the next prescription, we say that a gap occurs. In this state (**B**) **implementation behaviour** is expressed by events of alternating between being in a state of sufficient supply of medicine and in a state of a supply gap. A supply gap is defined as less than 51 days without medicine. If a supply gap exceeds 50 days, then we assume that (**C**) **treatment has been discontinued** by the patient and he or she is no longer persistent. Details on definition of these outcomes are described by Jensen et al.[18] based on a multistate model.

Since 1998, information on prescribed daily doses of medicine has been recorded in electronic medical records (EMR) at Steno. Data containing a unique identification number, date of birth, year of T2DM diagnosis, referral and discharge dates to and from Steno, and information on prescribed medicine were extracted from EMR. These data were transferred to Statistics Denmark and linked to the Register of Medicinal Product Statistics (RMPS) containing information at an individual level on prescription medicine sold at all Danish pharmacies since 1995.

Prescribed medicine (from EMR) and prescriptions redeemed by the patients (from RMPS), were coded according to the fifth level of the Anatomical Therapeutic Chemical (ATC) classification system.

### Covariates

Sex, country of origin, level of education, marital state and annual personal income at date of entry at Steno were used as covariates. Five time-varying covariates were included in the analyses: 1) age, 2) duration of T2DM diagnosis, 3) a Medication Complexity score (MC score): the number of medicines prescribed at any time during follow-up as an expression of polypharmacy, 4) Charlson’s Comorbidity Index (CCI) expressing the degree of comorbidity at any time during follow-up, and 5) number of times the patient has entered the state in question.

We used data from the Danish National Patient Register (NPR), which contains information on diagnoses at Danish hospitals since 1977 for calculation of CCI; the Danish Register of Causes of Death containing date of death; and other public registers accessible at Statistics Denmark containing information on migration, highest level of achieved education, marital state, income, and country of origin.

Level of education was categorised according to the International Standard Classification of Education (ISCED 2011). Country of origin was defined by Statistics Denmark’s classification: Danish origin, descendant or immigrant. Descendants and immigrants from the same country were pooled.

Current age and duration of diabetes were modelled as log-linear effects.

The CCI was calculated for each patient at entry to Steno. Registrations of diagnoses from the NPR from up to 10 years prior to entry date were classified into the 17 comorbidity groups in the CCI as described by Christensen et al.[19][20] and Green et al.[20]. Comorbidity group weights, updated and validated by Quan et al.[21], ranging between 0 and 6 points for each comorbidity group in the CCI were used. The NPR has used International Classification of Disease (ICD)-8 codes during 1977–1993 and ICD-10 codes since 1994. All diagnoses were coded to last for 10 years after date of diagnosis and then expire. For each day during follow-up and for each comorbidity group, we checked if new valid diagnoses were registered. If so, the weight of the comorbidity group to which the new diagnosis belonged was added to the total CCI for the patient in question from date of diagnosis and 10 years onwards.

MC score, CCI, and the number of times entering the state in question varied within each person during follow-up. Similar to age and duration of diabetes, we used the current value of the scores at the time of the left endpoints of the follow-up intervals.

### Calculations and statistical analyses

Implementation was calculated as the proportion of days with sufficient supply while being persistent[18]. It is inherent in the algorithm that the size of the implementation degree depends on both the maximum acceptable length of a supply gap and the prescribed amount of medicine, which at Steno is typically 3 months at a time. Persistence was calculated as the proportion of days in persistence of all days prescribed with the medicine in question.

Rate-ratios of incidence of the different events representing the different behavioural elements of adherence to medication over time were analysed through Poisson regression, where the follow-up time for each individual was split in intervals of 3 months, and one record for each time interval entered the Poisson model.

For the five time-varying covariates

1. age,
2. duration of T2DM diagnosis,
3. the Medication Complexity score (MC score): the number of medicines prescribed at any time during follow-up as an indicator of polypharmacy,
4. Charlson’s Comorbidity Index (CCI),
5. number of times the patient has entered the state in question,

we used the current value of the covariates at the time of the left endpoints of the follow-up time intervals. Thus, age and duration of T2DM diagnosis increased by 3 months for each follow-up time interval related to the same individual.

The Poisson regression models rates of each of the events, i.e. incidence of initiation of therapy, incidence of changing state from supply of medication to medication gap, incidence of changing state from medication gap to supply of medication, and incidence of discontinuation of medication treatment.

For the categorical variable “country of origin”, the incidence rate for individuals of Danish origin was used as reference to produce incidence rate-ratios between individuals of Turkish/Pakistani origin compared to individuals of Danish origin.

The effects of current age and duration of diabetes were entered as linear on the log-rate scale in order to have a parsimonious representation of the effect of these variables. For the four continuous covariates (age, diabetes duration, MC score, number of transitions out of state) we report the incidence rate-ratio by an increase of five years of age, of five years of diabetes duration, of 1 in MC score and of 1 in no. of transitions out of state, respectively. Thus all estimates for each transition rates are derived from the same Poisson model.

Calculations and analyses were performed using SAS, version 9.3 (SAS Institute Inc., Cary, NC, USA). Graphs were done in R, version 3.0.2 (http://www.R-project.org/).

### Ethics statement and regulatory approvals

Access to and use of data have been granted by the Danish Data Protection Agency (J.no. 2009-41-3056 and 2014-41-2819) and The Danish Health and Medicines Authority (J.no. 7-505-29-1553/1). All linked data was anonymised before being made available to us by Statistics Denmark. Written consent from patients was not needed.

## Results

A total of 5,232 patients (58% men) included in the study had been prescribed with 107 different medicines from the following ATC classes: blood glucose lowering medicine, excluding insulins (A10B), acetylsalicylic acid (B01AC06), antihypertensives (C02), diuretics (C03AB), beta blocking agents (C07), calcium channel blockers (C08), agents acting on the renin-angiotensin system (C09), and lipid modifying agents (C10). Thirteen medicines were prescribed to more than 500 patients [Table 1].

**Table 1:** Use of 13 most commonly prescribed medicines.

Metformin—A10BA02	3.449	66%
Glibenclamide—A10BB01	567	11%
Glimepiride—A10BB12	1.489	28%
Acetylsalicylic acid—B01AC06	3.856	74%
Bendroflumethiazide C03AB01	1.714	33%
Furosemide—C03CA01	1.627	31%
Metoprolol—C07AB02	1.004	19%
Amlopidine—C08CA01	1.588	30%
Enalapril—C09AA02	1.583	30%
Losartan—C09CA01	534	10%
Irbesartan—C09CA04	1.210	23%
Simvastatin—C10AA01	3.469	66%
Atorvastatin—C10AA05	746	14%

Number of patients (% of all 5,232 patients).

For analyses in this study, metformin (A10BA02), the frequently used groups of sulfonylureas (SUs) (A10BB), acetylsalicylic acid (B01AC06), thiazide diuretics in combination with potassium (C03AB), angiotensin receptor blockers (ARBs) and angiotensin converting enzyme inhibitors (ACEs) (C09AA+C09CA), and statins (C10AA) were selected. Insulins (A10A) were not included although they are often part of a rather complex blood glucose lowering medication regimen for T2DM [10,11]. As insulin doses are not always determined in advance and also dependent on physical activity and food intake, calculation of an adherence pattern for insulins is not possible.

With a median age of 59.5 years [IQR: 51.8–68.4] and a median DM duration of 6 years [IQR: 2–11] at entry, the patient population was followed for a total of 22,862 person-years (mean: 4.4 years [std: 4.1]). An additional 382 person-years spent in trials at Steno were censored. Patients were primarily of Danish origin. Patients of Turkish or Pakistani origin constituted the other two largest groups (3% + 3%). One in five had achieved tertiary level education and above, one third upper secondary, and one third lower secondary education, with a higher proportion of men at upper secondary and tertiary level. The men also surpassed the women’s annual income by €9,570. Two thirds of the patients were married, and of the remaining there were more widows than widowers. The majority of patients had prescriptions of two medicines or less at entry [Table 2]. At entry more than 88% had a CCI of either 0 or 1 [Table 2], indicating that less than two medical conditions existed simultaneously.

**Table 2:** Baseline characteristics.

At entry:		Men	Women	All
N		3.014	2.218	5.232
(%)		58%	42%	100%
				
Age, years (median)		59,5	61,2	60,0
[IQR]		[51.7–66.9]	[52.1–70.5]	[51.8–68.4]
				
DM duration, years (median)		6	6	6
[IQR]		[1–11]	[2–12]	[2–11]
				
Date of entry (median)		Oct.2002	Aug.2002	Oct.2002
[IQR]		[Jul.99 - Dec.05]	[Mar.99 - Jan.06]	[May.99 - Jan.06]
				
Country of origin		100%	100%	100%
	Denmark	85%	81%	83%
	Turkey	3%	4%	3%
	Pakistan	3%	4%	3%
	Other	9%	10%	10%
	Unknown	1%	1%	1%
				
Education		100%	100%	100%
	Lower secondary	30%	41%	35%
	Upper secondary	43%	32%	38%
	Tertiary and above	21%	15%	18%
	Unknown	6%	12%	9%
				
Marital status		100%	100%	100%
	Unmarried	15%	12%	14%
	Married	66%	55%	62%
	Divorced	13%	16%	15%
	Widow/Widower	4%	14%	8%
	Unknown	2%	2%	2%
				
Annual income, € (median)		30.483	20.913	24.979
[IQR]		[18,750–46,427]	[15,140–30,181]	[16,958–39,342]
				
				
MC score ^a^, median		1	1	1
[IQR]		[0–3]	[0–3]	[0–3]
	0	39%	40%	39%
	1	19%	19%	19%
	2	15%	14%	14%
	3	9%	10%	10%
	4	7%	7%	7%
	> = 5	11%	10%	11%
Total		100%	100%	100%
				
CCI		0	0	0
[IQR]		[0–1]	[0–1]	[0–1]
	0	60%	61%	60%
	1	29%	26%	28%
	2	6%	8%	7%
	> = 3	5%	6%	5%
Total		100%	100%	100%
				
Follow-up time, years (mean)		4,3	4,4	4,4
(std)		(4.0)	(4.2)	(4.2)
				

^a^ MC Score: Medication Complexity score; CCI: Charlson’s Comorbidity Index.

**Fig 1:**
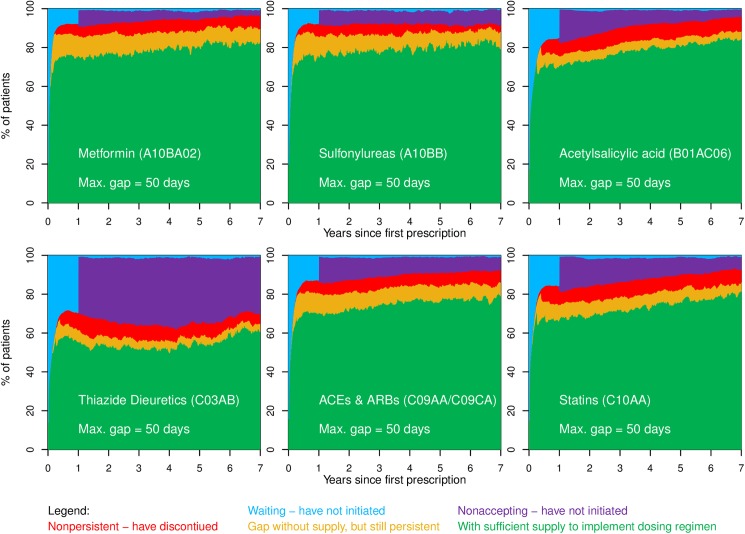
Time course of adherence to metformin, sulfonylureas, acetylsalicylic acid, thiazide diuretics, ACEs & ARBs, and statins. Patients are categorized in one of five different states during follow-up: Waiting and not having initiated treatment yet is represented by the blue area in the figure; having filled prescriptions and having sufficient supply of medication to cover the daily prescribed dose (green area); being without supplies for a maximum of 50 days (yellow area); having discontinued treatment after a gap of 50 days without supplies of medication (red area), whereas the purple area represents patients who do not initiate treatment by filling a prescription within the first 360 days and hence have not accepted the treatment. Patients will move between the 5 states during follow-up according to their adherence behavior. The proportion of patients in each of the 5 states at any given time is shown on the vertical axis. Further details are described by Jensen et al.[19].

Fig 1 shows unadjusted variations in the adherence pattern between the 6 selected medicine groups. During the first year of follow-up the proportion of patients not having initiated treatment varied from 12.7% [95%CI: 11.5–13.9%] for sulfonylureas to 35.3% [95%CI: 34.2–36.5%] for thiazide diuretics (blue + purple areas in Fig 1). The highest proportion of patients with sufficient supply (green area in Fig 1) during the first five years was seen for metformin (77.4% [95%CI: 77.2–77.6%]) and SUs (77.7% [95%CI: 77.5–78.0%]) and the lowest for thiazide diuretics (53.7% [95%CI: 53.6–54.0]). Once in treatment, the degree of implementation (the proportion of the combined green and orange areas being green in Fig 1) during years 2–5 was stable at a level around 89.5% [95%CI: 89.5–89.6%] for metformin to 94.4% [95%CI: 94.3–94.4%] for acetylsalicylic acid. During years 2 to 5 after the first prescription of medicine the proportion of patients having discontinued treatment (red area in Fig 1) were 5.0% [95%CI: 4.9–5.0%] for statins to 7.7% [95%CI: 7.6–7.8%] for thiazide diuretics.

### Age

**Fig 2:**
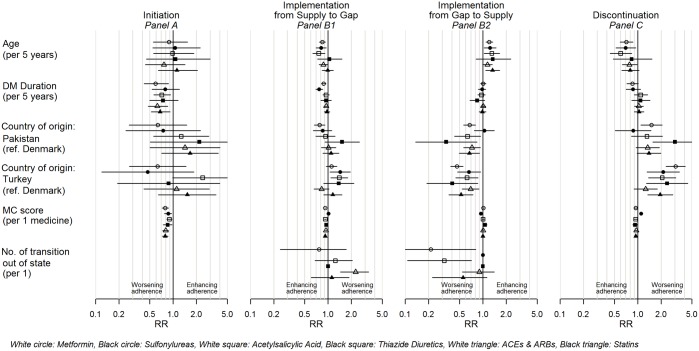
Rate-ratios for incidence of events of adherence to medication. Rate-ratios for Age (per 5 years), Diabetes duration (per 5 years), Country of origin with Denmark as reference, Medication Complexity score (per 1 unit), and Number of transitions out of state (per 1 transition). Panel A: For first initiation of treatment. For implementation: Panel B1: For running out of supply into a supply gap; Panel B2: For resuming supply of medicine after a supply gap. Panel C: For first discontinuation of treatment. Panels A–D show rate-ratios for metformin, sulfonylureas, acetylsalicylic acid, thiazide diuretics, ACEs & ARBs, and statins. DM, Diabetes Mellitus. MC score, Medication Complexity score. ACEs, Angiotensin Converting Enzyme inhibitors. ARBs, Angiotensin Receptor Blockers.

Younger age was associated with poorer adherence, as it was associated with gaps of supply (for metformin, SUs, and acetylsalicylic acid) [Fig 2B1]. Resuming supply of medicine after a supply gap was positively associated with older age for metformin, SUs, acetylsalicylic acid, and statins [Fig 2B2]. Furthermore, younger age was associated with discontinuation of metformin, SUs, and acetylsalicylic acid [Fig 2C].

### Duration of diabetes mellitus

Longer diabetes duration was significantly associated with lower incidence of initiation of metformin, acetylsalicylic acid, ACEs & ARBs, and statins [Fig 2A], whereas diabetes duration was inversely associated with running out of supply to a supply gap (for metformin and SUs) [Fig 2B1], indicating equivocal direction of the association between duration of diabetes and the different phases of adherence to medication.

### Country of origin

Turkish origin was positively associated with risk of supply gaps for SUs and acetylsalicylic acid [Fig 2B1]. Pakistani origin (all medicine groups except SUs) and Turkish origin (all medicine groups) were inversely associated with resuming supply of medicine after a supply gap [Fig 2B2]. Pakistani origin (for metformin and thiazide diuretics) and Turkish origin (all medicine groups except for ACEs and ARBs) were associated with discontinuation [Fig 2C]. Thus, all significant results were unequivocal towards an association between Pakistani/Turkish origin and poorer adherence compared to patients of Danish origin.

### Medication Complexity score

Higher MC score (a proxy for polypharmacy) was significantly associated with lower incidence of initiation for all medicine groups except thiazide diuretics [Fig 2A] thus impacting adherence negatively. But at the same time the MC score was negatively associated with running out of supply to a supply gap (all medicine groups except statins) [Fig 2B1], as well as being negatively associated with discontinuation (all medicine groups except statins) [Fig 2C].

### Number of transitions out of a state

The number of times spent in a state of being with supply of ACEs and ARBs was positively associated with running out of supply to a supply gap, meaning the more times a patient has been in and out of supply (e.g. experiencing a repeated outage of medication supply), the more likely the patient will run out of supply again [Fig 2B1]. Conversely, the number of times spent in a state of a gap for metformin and acetylsalicylic acid was inversely associated with resuming supply of medicine after a supply gap [Fig 2B2].

### Results across initiation, implementation and discontinuation

There was a small, but insignificant trend that women were more adherent to medication therapy than men (results not shown). Marital status, level of education, income, and burden of comorbidities (CCI) were not associated with neither initiation, implementation nor discontinuation of treatment (results not shown). An interaction between gender and being with or without a partner (based on a hypothesis that single men have a different risk profile than single women), was not significant (results not shown).

## Discussion

### Principal findings

The study showed that poor initiation, rather than poor implementation or discontinuation, was the biggest contributor to overall nonadherence. Use of a multi-state model made it possible to distinguish between the three different elements of adherence. By investigating the occurrence of four different behavioural events, our study adds to previous literature with this clear distinction between initiation, implementation and discontinuation. This gives an insight into the timing aspects of adherence that simple adherence rates or medication possession rates cannot[11]. Thus, we have been able to add details to the associations between adherence to medication and duration of diabetes and polypharmacy. Once the patients were in treatment, polypharmcay was not associated with poorer implementation, and it was inversely associated with early discontinuation, which to our knowledge are new findings.

### Strengths and limitations

Attempts to generalise the results from this study to other diabetes cohorts should be done with caution. It is estimated that approximately 20% of all T2DM patients in Denmark, primarily complicated patients, are referred to tertiary care[22]. The Steno population is representative of this group. The remaining approximately 80% are treated in primary care by general practitioners. It would be of interest to investigate if the primary care sector is able to achieve the same stable degree of persistence over time or if other patterns prevail. However, patients referred to tertiary referral hospitals are a very important segment of T2DM patients with high risk of further complications and a disproportionate high encumbrance on the health care system.

In Denmark the tax-funded nationwide healthcare system provides universal coverage to all residents. Prescription drugs are paid for by the patient, but extensive reimbursement options are available from the health authorities[23]. Income was not significantly associated with adherence to medicine in our study. It is possible that some patients, not eligible for extra individual reimbursement, were economically challenged, which might have reduced adherence to medication therapy. It was not possible to investigate the effects of individual reimbursement in this study. Studies have found that there is no significant inequity in use of all healthcare services in Denmark[24, 25]. But for services with co-payment, such as dental treatment and medicine some inequity appears to be present, disfavouring the lower income groups, immigrants and their descendants[24, 25]. Our study showed that supply gaps and discontinuing treatment were a bigger challenge for patients of Turkish/Pakistani origin compared to patients of Danish origin.

It should be noted that the study design has an inherent risk of representing a “survivor cohort” of patients retained in tertiary care over a longer period of time. This could partly explain the absence of signals of inequity with regard to income. It also emphasizes the need for analyses of the whole spectrum of T2DM patients.

Although not part of this study, analyses of adherence to insulins are also of high importance for this kind of study population. However, since calculations of adherence are challenged by frequent undetermined doses, and considerations of how to allow for waste of insulin, analyses of insulins were not performed. A review by Davies et al.[26] has also mentioned the poor quality of studies on adherence to insulin therapy due to the use of non-validated tools.

This study is based on registries containing information on both prescribed medication and what is actually filled, but without information from a Medication Event Monitoring System or similar pill count systems. This means we do not have information on which particular days the medicine is ingested if at all. Our data can tell when and how much medicine is available to the patient, and has the advantage of covering 22,862 person-years of follow-up. It would be an extremely comprehensive and expensive study if a daily pill count system were to be used for such a long period of follow-up time including a substantial number of different medicines.

Clinical parameters (e.g. blood pressure, HbA_1c_, or LDL cholesterol) from the patients were not included in the modelling of the rates of adherence because the major impact of these are most likely mediated through the prescription pattern already included in the model. We are very well aware of the challenges of time-dependent confounding because prescription of medication is dependent on previous measures of clinical parameters and a patient’s adherence to the prescribed medication is, too. At the same time, future measures of clinical parameters are dependent on previous patterns in prescription of medication and adherence to the prescribed medication.

In line with our study, Karter et al.[27] have emphasised the need for more attention to address nonpersistence in the very first stages after a new medicine is prescribed, which corresponds to the relatively large proportion of patients waiting to initiate treatment during the first year in our study. We were unable to investigate to which extent the period when patients were waiting to initiate treatment[23, 28]could be attributed to a switch between two medicines from the same class, where the patient was not to start the new medicine before any stock of the old medicine had been used.

Time during any hospitalisations was not accounted for. Beloin-Jubinville et al.[29] have found that hospitalisation did not appear to influence patient behaviour towards adherence to medication therapy. The extent to which the patients have been supplied with medication from a hospital during any hospitalization was not possible to determine.

Therapy related factors such as adverse effects, patient friendliness of regimen, dose-dispensed medicines[30] (also known as multidose drug dispensing[31]) vs. traditional pill boxes and blister packs, fixed-dose single-pill combinations[32], and taste of the medicines[33] were not investigated.

### Findings in relation to other studies

Guénette et al.[34] reported persistence and implementation to be associated with older ages and a history of using 5 or more medicines, which supports our results. Lemstra et al.[16] found immediate discontinuation after a single fill contributes disproportionately to statin nonadherence in three Canadian cohorts. Hansen et al.[35] detected a large group of “early quitters” (premature medication therapy discontinuation with ≤ 84 DDD during the first 6 months) in a study of antiosteoporotic therapy among 100,949 Danish men and women. Both Blaschke et al.[12], from a database including 95 clinical studies, and Yeaw et al.[36], from a pharmacy claims database, reported a large proportion of patients discontinuing treatment within the first year. This picture of discontinuation is not seen in our study, perhaps due to the setting of Steno as a tertiary referral hospital with frequent interactions between patient and health care professionals, close monitoring of patients, and the before mentioned inherent risk of a “survivor cohort”.

Conflicting results for diabetes treatment are found by Rolnick et al.[37]. They found higher adherence in men and in those on fewer medicines (similar to our MC score). In a Danish study by Wallach-Kildemoes et al.[38], adherence to preventive statin therapy decreased with increasing income. The two Danish cohorts are probably not comparable (predominantly primary vs. tertiary care, and patients with no prior diabetes diagnosis vs. T2DM patients with an overrepresentation of patients with a high disease burden). In an Australian cohort of statin users, Warren et al.[39] discovered a trend towards greater adherence (in terms of medication possession ratio) with increasing age. Patients with a history of prior heart disease were more likely to adhere to long-term use of statins. Language other than English was a major risk factor for non-adherence. If language barriers can be assumed to pose a challenge for a majority of the Steno patients of Turkish/Pakistani origin, our findings on age and ethnicity are in line with Warren’s.

The CCI was included in the analysis as a proxy for comorbidity. It did not show any associations with adherence to medication therapy. Diagnoses of depression are not included in CCI. However, depression has independently been associated with lower adherence among patients with chronic obstructive pulmonary disease[40] and in hypertensive patients[41]. Cross-sectional studies have shown associations between patients’ illness perception and adherence to medication therapy[42–44]. Future studies of associations between comorbid conditions and adherence to medication therapy may consider depression and patients’ illness perceptions and beliefs.

A translation of the findings to clinical management of diabetes would focus on one major element: An effort to support patients further when they are introduced to new drug combinations/complex drug strategies should be prioritized since inertia was seen in initiating treatment, and medication complexity (a proxy for polypharmacy) as well as duration of diabetes were risk factors for poor initiation of treatment. The proportion of persistent patients was stable over time and there were no increase in supply gaps or discontinuation over time, which further supports the view of prioritizing initiation of treatment. This should be seen in the context of a tertiary care setting with frequent interactions between patient and health care professionals and a close monitoring of patients. Furthermore, intervention studies are needed to test methods, particularly for improving initiation of further amendments to medication therapy among patients already on a complex medicine regimen.

## Abbreviations

ACEs: Angiotensin Converting Enzyme inhibitors
ARBs: Angiotensin Receptor Blockers
ATC: Anatomical Therapeutic Chemical classification system
CCI: Charlson’s Comorbidity Index
DDD: Defined Daily Dose
DM: Diabetes Mellitus
EMR: Electronic Medical Records at Steno Diabetes Center
ICD: International Classification of Diseases
IR: Incidence Rate
MC score: Medication Complexity score
MPR: Medication Possession Ratio
NPR: Danish National Patient Register
RMPS: Register of Medicinal Product Statistics
Rx: Filled Prescription redeemed by patient
Steno: Steno Diabetes Center
SUs: Sulfonylureas
T2DM: Type 2 Diabetes Mellitus
